# Caution: Wit and Humor During the COVID-19 Pandemic

**DOI:** 10.1177/1071100720923651

**Published:** 2020-04-26

**Authors:** Christopher P. Chiodo, Kimberly K. Broughton, Max P. Michalski

**Affiliations:** 1 Brigham and Women’s Hospital, Boston, MA, USA; 2 Harvard Medical School, Boston, MA, USA

If there ever was a time in our lives that we could use a smile or good laugh, it’s probably now. Thousands have died, and each day, the headlines grow more bleak as the COVID-19 pandemic progresses. Hopefully, the curve can be flattened and lives saved. This process will take time, however, and as such, we are likely facing a prolonged crisis that will last several months.

In the setting of this unfolding calamity, we are faced with so many critical questions. One that might be easily overlooked is the current role of humor in our professional lives. The importance of this question derives as much from our roles as team leaders, as it does our status as physicians.

Given the potential for humor to relieve tension and settle frayed nerves, is now a better time than any to offer a witty comment to a colleague or your team? On the other hand, might that same witticism be frowned upon given our temporal and physical proximity to so many sick and dying individuals?

While this may feel a bit like standing in front of the Oracle at Delphi, the 2 positions may not be so diametrically opposed and can be reconciled with some careful thought. More specifically, it may be possible to still employ wit and humor but with some important checks employed. Yes, that fatigued and stressed colleague could certainly benefit from a smile. However, there are some new considerations that must be taken into account while trying to brighten a moment during the current crisis. In formulating the principles delineated below, we’ve reached out to some brave and inspiring colleagues who are at the front lines of the current pandemic.

*Tone it down, but don’t turn it off.* Last week, there was palpable tension among the receptionists and medical assistants in our office. After a few words of encouragement and thanks, there seemed to be something missing. Clinics are generally lighthearted and fun. After a quick joke about the poor ham sandwich who couldn’t get a beer at the bar because the bartender said he doesn’t serve food, the tension eased. Everyone smiled, a few laughed out loud, and one person went out of her way to say thanks. For the rest of the day, though, it was a more somber environment, appropriately so. After all, there is always the risk of being considered silly if humor is used too frequently. That rule is even more important now, and the “silliness threshold” is likely lowered. One nurse from the hard-hit Los Angeles region reported, “Humor is still alive in our ICU. It helps unify us, makes you feel you are a part of a team, and not so isolated in a lonely time. . . . As a member of a pretty close knit team I feel that making jokes is part of my job. I have not let up . . . I know it lightens the heaviness we feel in our chests on a daily basis. But when things hit the fan, we all work hard. No joking during that time.”

*Always try to laugh with, and not at, someone.* Professor Rod Martin is a clinical psychologist from Canada who is an authority on the benefits of humor. Countless schemes have been derived to classify humor, of which his is one of the most well known. He divided humor styles as either being benign or injurious.^2^ Along these lines, it is generally safer to laugh with each other, rather than at someone. Laughing at someone invokes the superiority theory of humor, which is socially dangerous, harmful, and detrimental to teams.

The exception to this rule, however, is the use of self-defeating humor. While this type of humor may not always fit into the workplace, showing self-vulnerability in challenging times may ease the tension of others around you. Because self-defeating humor may be interpreted as ineptitude by a patient or anxious family, it is best reserved to be used among colleagues as a means of lightening a moment and enhancing your relationship with the team.

*Gallows humor is okay; just be careful.* Gallows humor includes jokes, irony, and humorous remarks about frightening topics such as combat and death. An active pandemic presents ample opportunity for the use of this form of humor. For some, gallows humor is a coping mechanism that may also boost camaraderie and morale.

However, it may also portray a clinical scenario as more grim than it really is. Joking that we’re all going to die from COVID-19 is obviously a gross overexaggeration and one that may increase anxiety among less informed members of a team. Another danger of gallows humor is that it may be overheard by an “outsider” who is offended, threatened, or caused to unnecessarily worry about a situation. Imagine an inpatient overhearing health care providers employing gallows humor about increased workload or a bleak prognosis.

When we spoke with an active emergency medicine physician who has also led previous global disaster responses, she commented that “gallows humor may actually be helpful when used carefully, and front-line providers shouldn’t have to feel guilty about using it. You just have to understand that it has a time and place.”

*Make it quick and short.* The members of a care team may be increasingly busy and stressed and may not have the time or emotional bandwidth to digest a long anecdote or complex joke. Irony, witty comments, and even “dad” jokes may represent simple aliquots of humor and brevity that will be just enough to brighten the moment without crossing the silliness threshold.

Quips and carefully placed momentary sarcasm can also be quick ways to bring about a smile or laugh. Remarks that take a surprise twist or unexpected perspective can get others out of mental ruts that occur during a crisis. The comments others “didn’t see coming” can be used to positively derail a dreary situation.

*Use technology to spread smiles.* With the current pandemic, we are more isolated than ever before. This can obviously take a toll on our psychological wellness and sense of community. Social media and electronic platforms offer a solution to this problem. To this end, members of younger generations commonly share humor and laughter electronically. For these individuals, memes and video segments have replaced witty dad jokes. Again, caution must come into play. Social media have permanency, and what one shares online can be seen by so many and even end up on the front page of the local newspaper.

*Mix in other leadership tools.* Humor is just one item in the toolbox of an effective leader. Now is the time to augment it with larger doses of other warmhearted interventions, such as expressions of gratitude and encouragement. A recent communication from the American Medical Association^1^ offered advice on addressing physician stress during the pandemic. One strategy was for leaders to “show compassion and empathy about what it’s like to be on the front lines.” Such gestures should be directed not only to physicians and nurses treating COVID-19 patients in the hospital but also to support staff who may be worried about furloughs and financial stress at home. Members of your team may have spouses and partners who have recently been laid off and have limited savings to weather the storm. For them, a few words of thanks or encouragement can go a long way.

*Laugh and decompress outside of the workplace.* Our team used to routinely go out for drinks at the end of the workday each Friday. Here we decompressed and laughed together. Humor abounded in this setting and fostered team cohesion. Unfortunately, such gatherings are now prohibited. On the bright side, a commercial videoconferencing platform allows us to still have weekly “virtual wine and cheese” gatherings. Despite the changes in our lives, we still smile and laugh, although probably not quite as much as we did before COVID-19. More important, though, we are together again in a relaxed and nonclinical “environment.”

With teamwork and fortitude, we will get through the current crisis and grow stronger for it. Until then, there will certainly be some challenging times. Smiles and laughter can hopefully brighten those moments, ease our stress, and help us get through this together. As one emergency room nurse concluded, “Humor absolutely relieves tension because it allows us all to laugh, decompress, and bond.”

## Supplemental Material

FAI923651_ICMJE_disclosures – Supplemental material for Caution: Wit and Humor During the COVID-19 Pandemic Click here for additional data file. Supplemental material, FAI923651_ICMJE_disclosures for Caution: Wit and Humor During the COVID-19 Pandemic by Christopher P. Chiodo, Kimberly K. Broughton and Max P. Michalski in Foot & Ankle International
